# Short- and long-term prognostic value of hyponatremia in patients with acute coronary syndrome: A systematic review and meta-analysis

**DOI:** 10.1371/journal.pone.0193857

**Published:** 2018-03-02

**Authors:** Qiang-Qiang Ma, Xiu-De Fan, Tao Li, Yuan-Yuan Hao, Feng Ma

**Affiliations:** 1 Department of Cardiology, Xi'an Central Hospital, Xi’an, Shaanxi Province, China; 2 Department of Infectious Diseases, the First Affiliated Hospital of Xi’an Jiao Tong University, Xi’an, Shaanxi Province, China; Azienda Ospedaliero Universitaria Careggi, ITALY

## Abstract

Hyponatremia is relevant to heart failure, liver cirrhosis and stroke, but the prognostic value of serum sodium levels in patients with acute coronary syndrome are still unclear. So we did a systematic review and meta-analysis to assess the prognostic value of hyponatremia on adverse events in patients after ACS. We systematically searched PubMed, Embase and Cochrane Library to find literatures which studied the prognostic value of hyponatremia in patients with ACS. Our main endpoints were the all-cause mortality and heart failure in the short- and long-term. Of 369 identified studies, 20 studies were included in our analysis. Compared with the normal natrium, hyponatremia was significantly associated with the increased risks of all-cause mortality within 30 days (RR: 2.18; 95%CI: 1.96–2.42) and during the follow-ups (HR: 1.74; 95%CI: 1.56–1.942). For the second endpoint of short- and long-term heart failure, the pooled effect sizes in hyponatremia patients were 1.72(95%CI: 1.38–2.14) and 1.69(95%CI: 1.12–2.55) respectively. In conclusion, hyponatremia has a significant prognostic value for short- and long-term adverse event in patients after ACS, the dynamic monitoring of serum sodium levels may could help physicians to identify high risk ACS patients and to stratify risk for optimal management.

## Introduction

Cardiovascular disease (CVD), especially acute coronary syndromes (ACS), is common in the general population, affecting the majority of adults past the age of 60 years. In 2012 and 2013, CVD was estimated to result in 17.3 million deaths worldwide on an annual basis [1–3]. Unstable angina (UA), acute non-ST elevation myocardial infarction (NSTEMI), and acute ST elevation myocardial infarction (STEMI) are the three presentations of ACS. Hyponatremia, defined as a serum sodium concentration ([Na^+^]) <135 mmol/L, is the commonest electrolyte disorder encountered in clinical practice. Previous studies had found that hyponatremia is closely related to the prognosis of heart failure [4, 5], stroke [6, 7], liver cirrhosis [8]and chronic kidney disease (CKD) [9, 10]. The underlying mechanism may be relevant to the release of vasopressin, activation of the renin-angiotensin system and catecholamine production [11, 12]. Furthermore, previously conducted studies had reported the prevalence of hyponatremia in patients with myocardial infarction ranges from 12.5%–23.2% [13], and some correlations had found in hyponatremia and the prognosis of myocardial infarction[13–32]. However, those individual studies have yielded inconsistent findings, possibly caused by limitations associated with individual studies. To shed light on the relationship between hyponatremia and the adverse outcomes of acute coronary syndrome and to more precisely evaluate the prognostic value of hyponatremia in patients with acute coronary syndrome, we performed a meta-analysis of published studies.

## Methods

### Data sources

The methods of this systematic review and meta-analysis were performed in accordance with the Preferred Reporting Items for Systematic Reviews and Meta-Analyses (PRISMA) Statement and it was registered at International Prospective Register of Systematic Reviews (numberCRD42016032836)[33].A comprehensive literature search was performed up to October 1,2017,without restriction to regions, publication types or languages. The primary sources were the electronic databases of PubMed, Embase and Cochrane Library, using various combinations of Medical Subject Headings (MeSH) and non-MeSH terms: "Hyponatremia" combined with "Myocardial Infarction”, "Myocardial Infarct", "Acute Coronary Syndromes", "Cardiovascular Stroke" and "Heart Attacks". The full electronic search strategy for PubMed was that ((“Hyponatremia”[Mesh] OR(Hyponatremias)) AND (“Myocardial Infarction”[Mesh]OR(Infarction, Myocardial)OR(Infarctions, Myocardial)OR(Myocardial Infarctions)OR(Cardiovascular Stroke)OR(Cardiovascular Strokes)OR(Stroke, Cardiovascular)OR(Strokes, Cardiovascular)OR (Heart Attack)OR(Heart Attacks)OR(Myocardial Infarct)OR(Infarct,Myocardial)OR(Infarcts,Myocardial)OR(Myocardial,Infarcts)OR(AcuteCoronarySyndromes)OR(CoronarySyndrome,Acute)OR(CoronarySyndromes,Acute)OR(Syndrome,AcuteCoronary)OR(Syndromes,AcuteCoronary))) The reference lists of all selected publications were checked to retrieve relevant publications that were not identified in the computerized search. The main search was completed independently by investigators. Any discrepancy was solved by consultation of an investigator, not involved in the initial procedure.

### Study selection and data extraction

Studies were included if they met the following criteria: (1) used a well-defined cohort design; (2) clearly stated hyponatremia as a major exposure in patients with ACS; (3) presented hazard ratio (HR) or relative risk(RR) for main outcomes with a 95% confidence interval (CI) or reported sufficient data to calculate these parameters. Exclusion criteria were as follows: (1) Patients with known heart failure, cirrhosis, pseudo-hyponatremia, hypothyroidism, adrenal insufficiency, malignancy, recent surgery within 1 month and those dying or discharged within 48 h; (2) Case reports, case series, review articles and abstracts;(3) insufficient information concerning evaluation rates.

One investigator abstracted data from all included studies into a standardized evidence table. The following information and data were extracted: name of the first author, publication year, study design, sample size, study population, duration of follow-up, country, main outcomes and HR or RR with 95% CI. The main outcomes included in-hospital mortality and all-cause mortality within 30 days (short-term mortality), all-cause mortality during follow-ups, in-hospital heart failure and readmission for heart failure during follow-ups. For studies with insufficient information, the investigator contacted the primary authors, when possible, to acquire and verify the data. A second investigator checked these data for accuracy. Disagreements were resolved by discussion or consensus with a third investigator.

### Statistical analysis and quality assessment

We used the relative risks (RRs) with 95% CIs to evaluate the prognostic value of hyponatremia in ACS during the short-term, for analyzing the association between hyponatremia and the long-term adverse events, HRs with 95% CIs were assessed. When the prognosis was presented only as the Kaplan-Meier curves in some studies, the Engauge Digitizer V4.1 was used to obtain the survival data, and Tierney’s method to calculate the HRs and 95%CIs[34]. Statistical heterogeneity among studies will be evaluated with Q and I-squared statistics [35, 36]. Sensitivity analysis will be performed to evaluate the stability of the results. If there was heterogeneity between the studies, summary HR or RR estimates and 95% CIs will be calculated using the method of DerSimonian and Laird by a random-effects model. A fixed effects model will be used for the statistical pooling of the data in the case of no heterogeneity among the studies. An estimation of potential publication bias will be executed by the funnel plot. If the funnel plot is asymmetrical, we will assess the data using Egger’s linear regression test[37]. All statistical tests will be performed with the STATA12.0 software. All statistical tests are two sided, P<0.05 is considered statistically significant.

Individual study quality will be judged using the Newcastle-Ottawa Scale(NOS)[38],which consists of three quality parameters: selection (0–4 points), comparability (0–2 points), and outcome assessment (0–3 points). The scores range from 0 to 9, with scores 0–4 indicating low quality and scores 5–9 indicating high quality. Two investigators independently performed this quality assessments.

## Results

### Literature search and study characteristics

**Fig 1:**
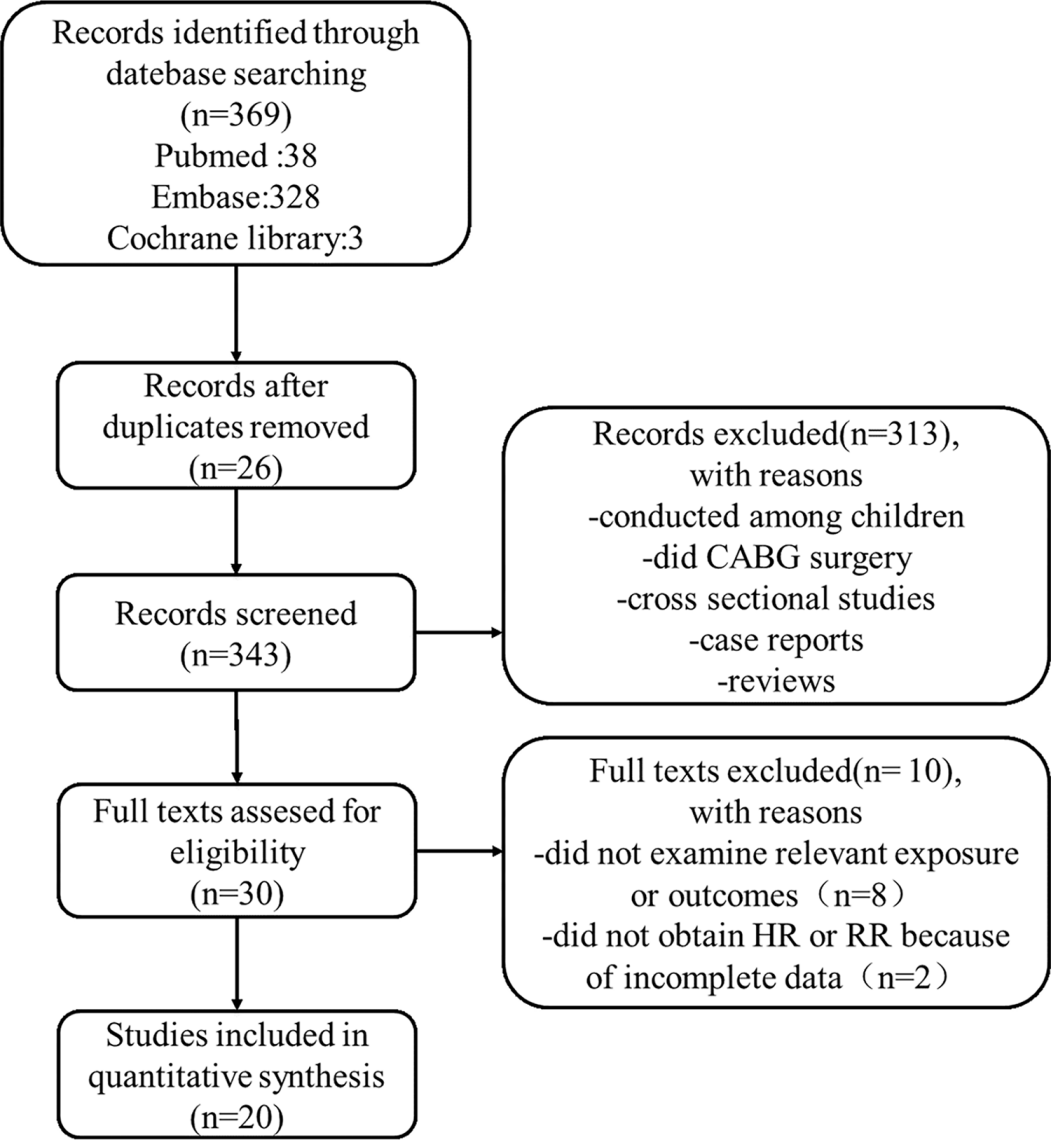
Flow chart of the selection process.

Overall, the systematic search of the databases revealed 369 publications for possible inclusion. Following the removal of duplicates, the remaining titles and abstracts were reviewed and irrelevant publications were excluded, mainly because they were reviews, cross sectional studies, case reports, conducted among children and did coronary artery bypass grafting(CABG) surgery. And then 30 publications left were reviewed in their entirety. Of those, 10 were excluded after more detailed inspection of full texts. We finally identified 20 publications[13–32], including 34,782 patients, which met inclusion criteria. No additional articles were added from manual review of the references. The flow chart of literature search and detailed exclusion reasons were shown in Fig 1 which based upon the PRISMA flow diagram for systematic review[39].

Baseline characteristics of 20 studies[13–32] included are listed in Table 1. 8 of those are prospective cohort studies[13, 14,18,21,23,24,26,32], others are retrospective cohort studies. The studies were conducted in the following countries: the United States, Israel, China, Indian, Korea, Poland, Czech, Denmark, Japan, Turkey, Italy and Germany. All the studies enrolled both men and women, the age ranged from 25 to 93. The median follow-up time varied from 30days to 18 years. Of 20 studies, 10 studies enrolled STEMI patients, 8 studies enrolled STEMI and NSTEMI patients, one study enrolled individuals with NSTE-ACS, and one study enrolled myocardial infarction patients with a low LVEF (EF≤35%) but not heart failure. All available studies used a composite reference standard based on the contemporary universal definition to diagnose myocardial infarction [40]. In terms of the main outcomes, 12 studies reported in-hospital mortality or all-cause mortality within 30 days, 4 reported in-hospital heart failure, 10 reported all-cause mortality during follow-ups and 2 reported the readmission for heart failure during follow-ups. As for study quality assessment, these 20 included studies were of relatively high methodological quality with their Newcastle-Ottawa Scale (NOS) scores ranging from 5 to 8.

**Table 1:** Characteristics of included studies on association between hyponatremia and clinical outcomes.

Study	Location	Study Subject	Sample Size	Follow-up	Outcomes	Study Quality
Goldberg 2004	Haifa, Israel	STEMI	1047	30days	all-cause mortality within 30 days	7
Goldberg 2006	Haifa, Israel	STEMI without HF	978	31(9–61)months	1.all-cause mortality during follow-ups 2.readmission for HF during follow-ups	7
Wang,L.F.2006	Harbin, China	AMI	670	NG	all-cause mortality within 30 days	6
Singla 2007	Pennsylvania US	NSTE-ACS	1478	NG	all-cause mortality within 30 days	8
Klopotowski 2009	Warsaw, Poland	STEMI	1858	NG	1.in-hospital mortality 2.in-hospital heart failure	8
Aziz, F. 2011	Jersey City, US	STEMI&NSTEMI	128	NG	in-hospital mortality	6
Havranek,S. 2011	Prague, Czech	STEMI	218	39±21months	all-cause mortality during follow-ups	7
Schou,M. 2011	Hillerod, Denmark	AMI&EF≤35	1731	17(16–18)years	1.all-cause mortality within 30 days 2.all-cause mortality during 1 year	7
Tada,Y. 2011	Saitama, Japan	STEMI	140	920days	1.in-hospital heart failure 2.readmission for HF during follow-ups 3.all-cause mortality during follow-ups	8
Tang,Q. 2011	Beijing, China	STEMI	1620	NG	1.in-hospital mortality 2.in hospital heart failure	8
Bozbay,M. 2012	Kastamonu, Turkey	STEMI	366	NG	in-hospital mortality	6
Lazzeri,C. 2012	Florence, Italy	STEMI	1231	NG	1.in-hospital mortality 2.all-cause mortality during follow-ups	7
Qureshi,W. 2013	Michigan, US	STEMI&NSTEMI	11562	5.5±3.3years	1.all-cause mortality within 30 days 2.all-cause mortality during follow-ups 3.congestive heart failure-related 30 day rehospitalization.	7
Harsoor S. 2014	Gulbarga, Indian	STEMI	100	NG	1.in-hospital mortality 2.in-hospital heart failure	8
Merchant,B.C. 2015	Worcester, US	ACS	2081	NG	all-cause mortality during 1 year	7
Burkhardt,K. 2015	Augsburg, Germany	STEMI&NSTEMI	3558	6(4.0–8.2)years	all-cause mortality during follow-ups	8
Plakht,Y. 2015	Beer-Sheva, Israel	AMI	2763	8.2years	all-cause mortality during follow-ups	8
Bae,M.H. 2017	Daegu, Korea	AMI	1290	12months	all-cause mortality during 1 year	7
Choi,J.S. 2017	Gwangju, Korea	AMI	1863	3.72±1.86years	all-cause mortality during 3 years	7
Devi,K.B. 2017	Imphal, Indian	STEMI	100	NG	in-hospital mortality	5

NG, not given; AMI, acute myocardial infarction; STEMI, ST-segment elevation myocardial infarction; NSTEMI, non-ST-segment elevation myocardial infarction; ACS, acute coronary syndrome; NSTE-ACS, non-ST-segment elevation acute coronary syndrome; HF, heart failure; EF, ejection fraction.

### Hyponatremia and short-term mortality in ACS

**Fig 2:**
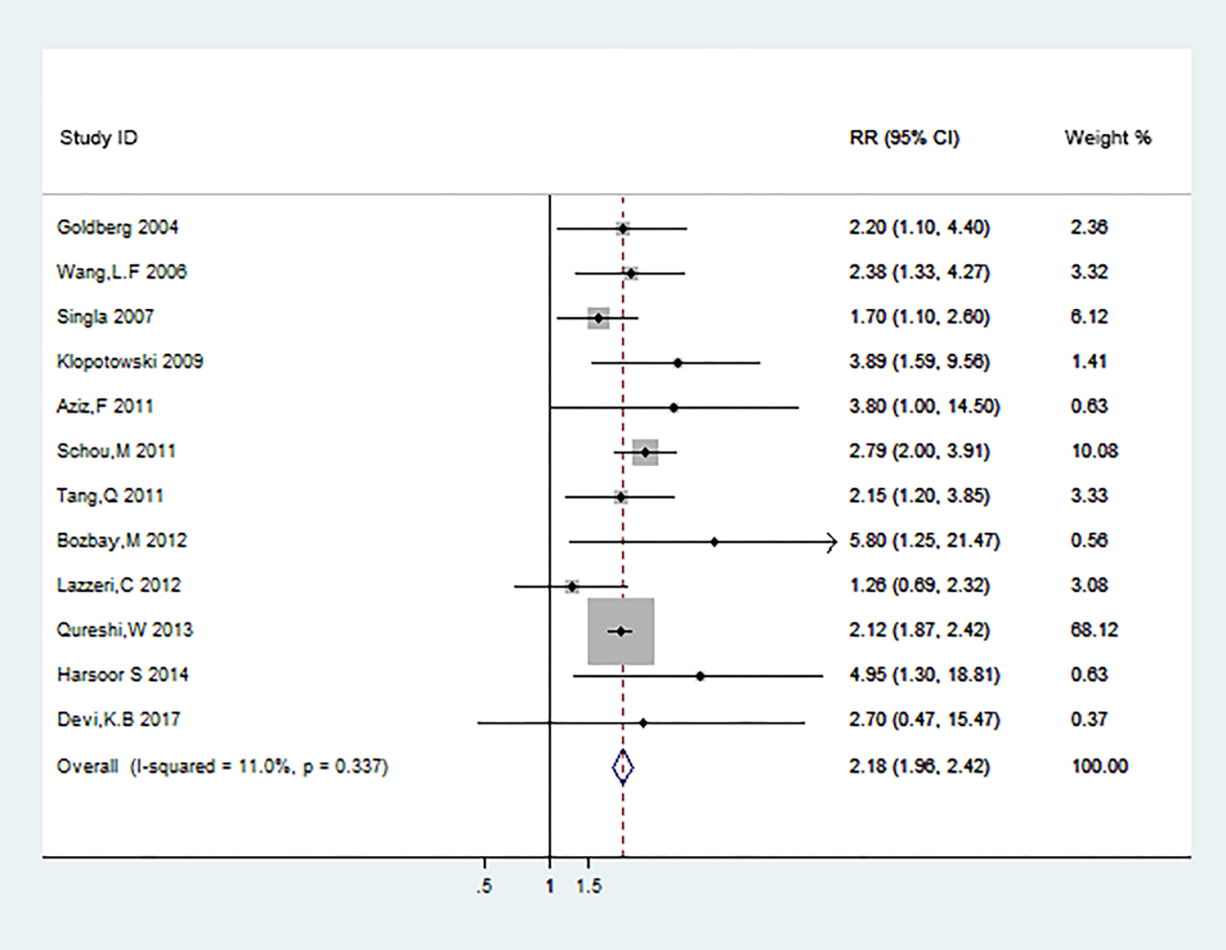
In-hospital mortality and all-cause mortality within 30 days.

**Fig 3:**
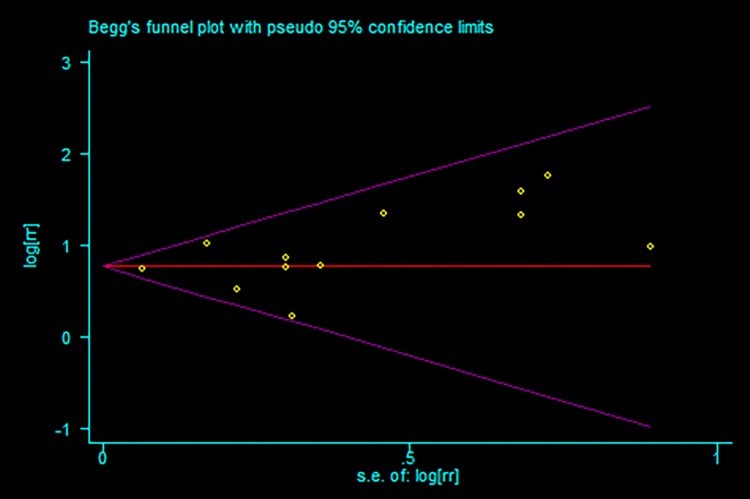
Funnel plots of studies included in the meta-analysis for short-term mortality.

12 studies [13,15–18,20,22–26,32] were included in the meta-analysis for the association of hyponatremia in acute coronary syndrome patients with the risk of short-term mortality, which included in-hospital mortality and all-cause mortality within 30 days. The relative risks for this association varied from 1.26 to 5.80 among 12 studies. Overall, patients with hyponatremia compared with the normal group experienced a significantly increased risk for short-term mortality (RR: 2.18 [95% CI: 1.96 to 2.42]; P<0.001) (Fig 2). Potential evidence of significant heterogeneity was not detected (P = 0.337; I^2^ = 11.0%). Publication bias was assessed among studies of hyponatremia and short-term mortality risk by using the Begg rank correlation test and Egger linear regression test, which did not suggested the publication bias (Begg, P = 0.244; Egger, P = 0.212; Fig 3).

**Fig 4:**
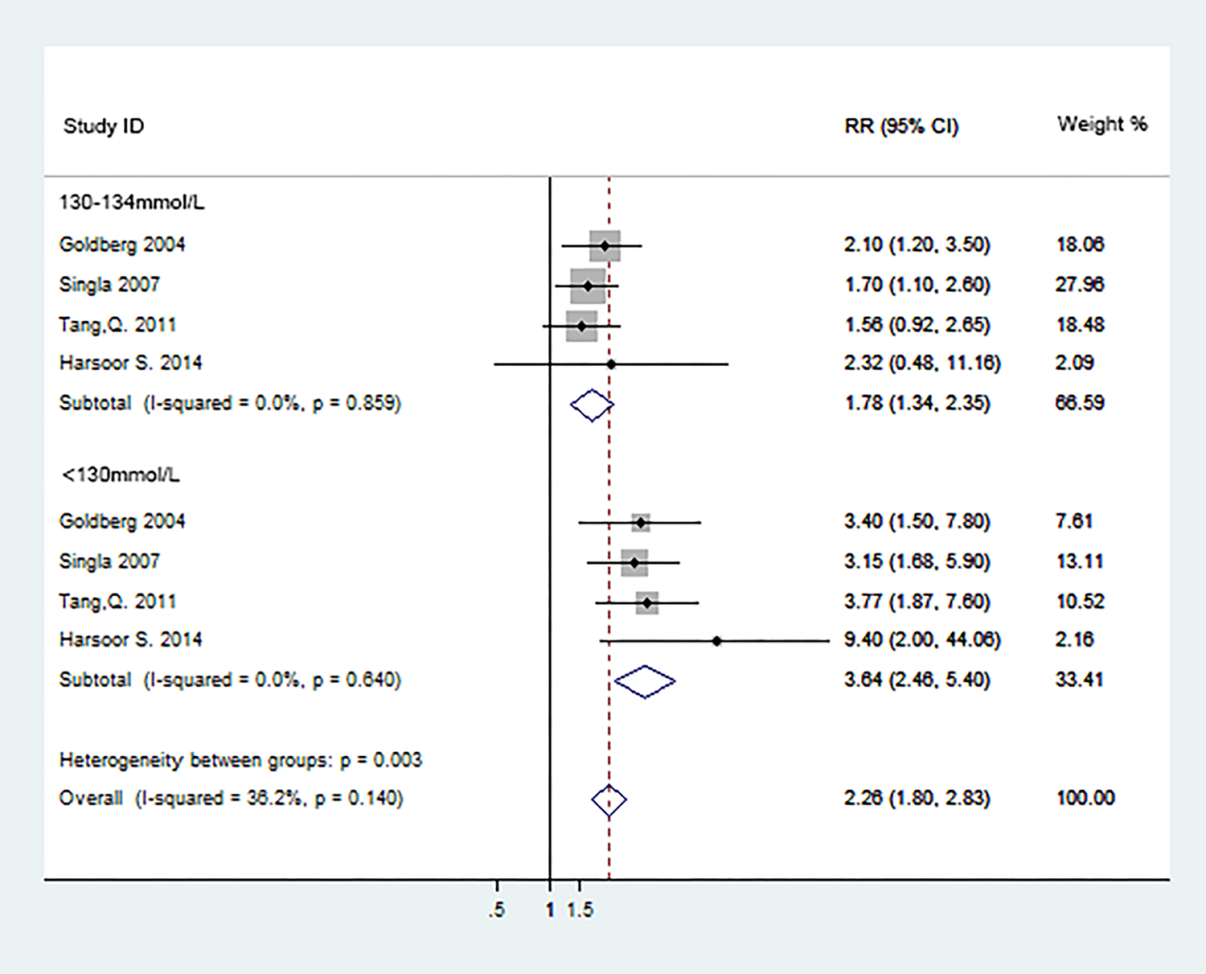
Subgroup analyses of short-term mortality according to serum Na+ levels.

Subgroup analyses were conducted according to the serum Na^+^ levels(130-134mmol/L and <130mmol/L).Of 20 studies, 4 studies[13,16,22,26] reported effect estimates on the serum sodium levels with short-term mortality, the analyses of subgroups were provided in Fig 4. The pooled RRs in relation to 130-134mmol/L were 1.78 (95%CI: 1.34–2.35; P<0.001).The incidence of short-term mortality was significantly increased by 364% (RR: 3.64; 95%CI: 2.46–5.40; P<0.001) in patients with the serum sodium levels<130 mmol/L. The incidence of the short-term mortality between two subgroups showed statistical significance (p = 0.003) and the subgroup of sodium levels<130 mmol/L showed higher short-term mortality than 130-134mmol group. The overall pooled RR was 2.26 (95%CI: 1.80–2.83; P<0.001).All subgroup analyses above did not show potential evidence of heterogeneity (P = 0.859, I^2^ = 0.0%; P = 0.640, I^2^ = 0.0% and P = 0.140, I^2^ = 36.2%) and no evidence of publication bias was detected (Begg, P = 0.063; Egger, P = 0.053).

### Hyponatremia and long-term mortality in ACS

**Fig 5:**
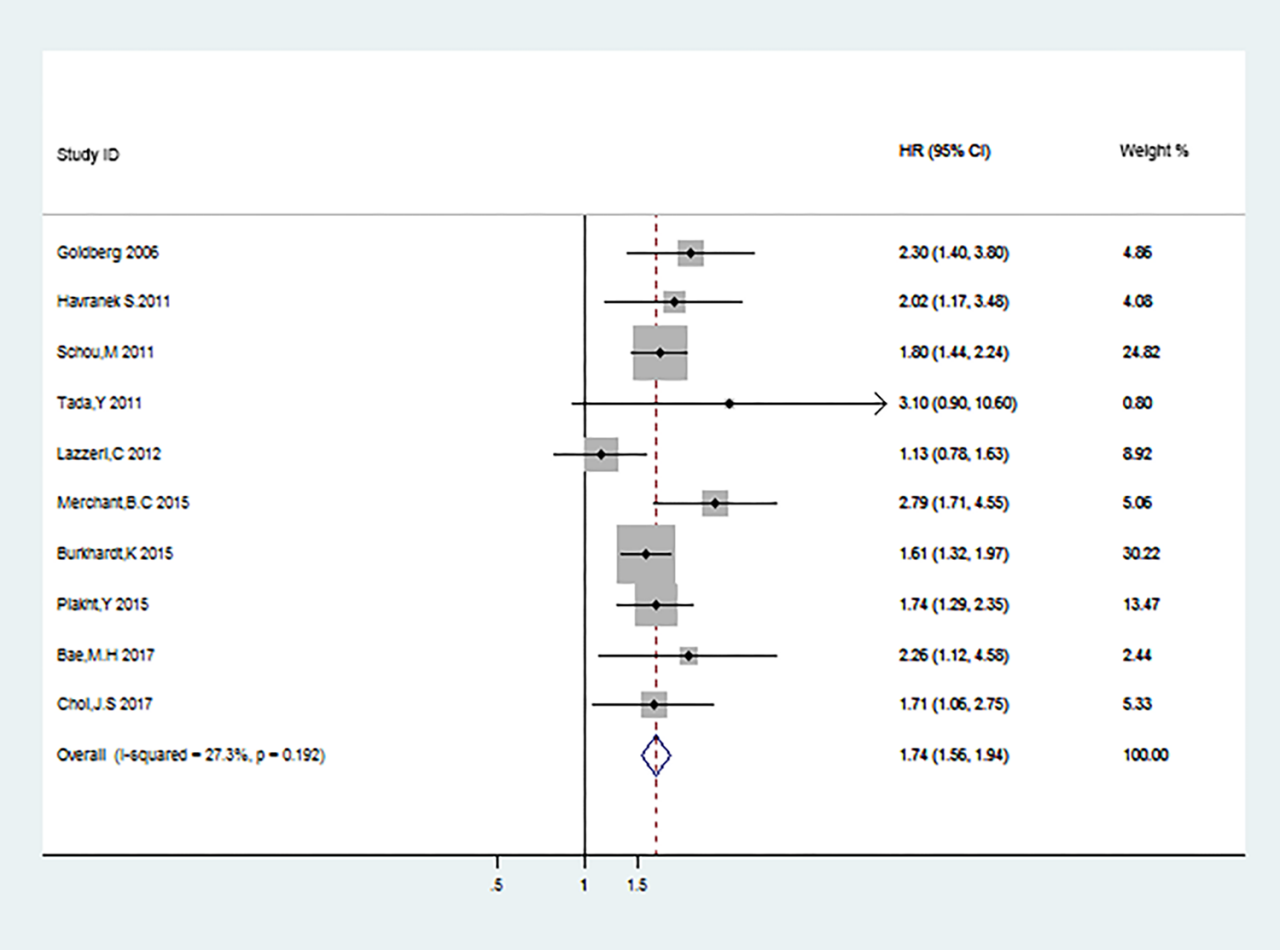
All-cause mortality during the following-ups.

**Fig 6:**
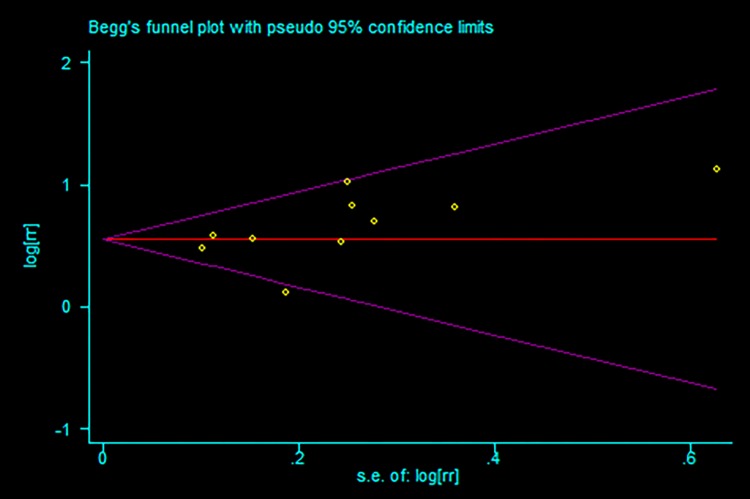
Funnel plots of studies included in the meta-analysis for long-term mortality.

Data from 10 studies [14, 19–21, 24, 27–31] were used to evaluate the effect of hyponatremia on long-term mortality in acute coronary syndrome patients. The pooled HR indicated that there was significant difference between hyponatremia and the normal natrium group on long-term mortality (HR: 1.74; 95%CI: 1.56–1.942; P<0.001, Fig 5) with no evidence of publication bias (Begg, P = 0.107; Egger, P = 0.164; Fig 6) and no statistically significant heterogeneity (P = 0.192, I^2^ = 27.3%).

### Hyponatremia and heart failure in ACS

**Fig 7:**
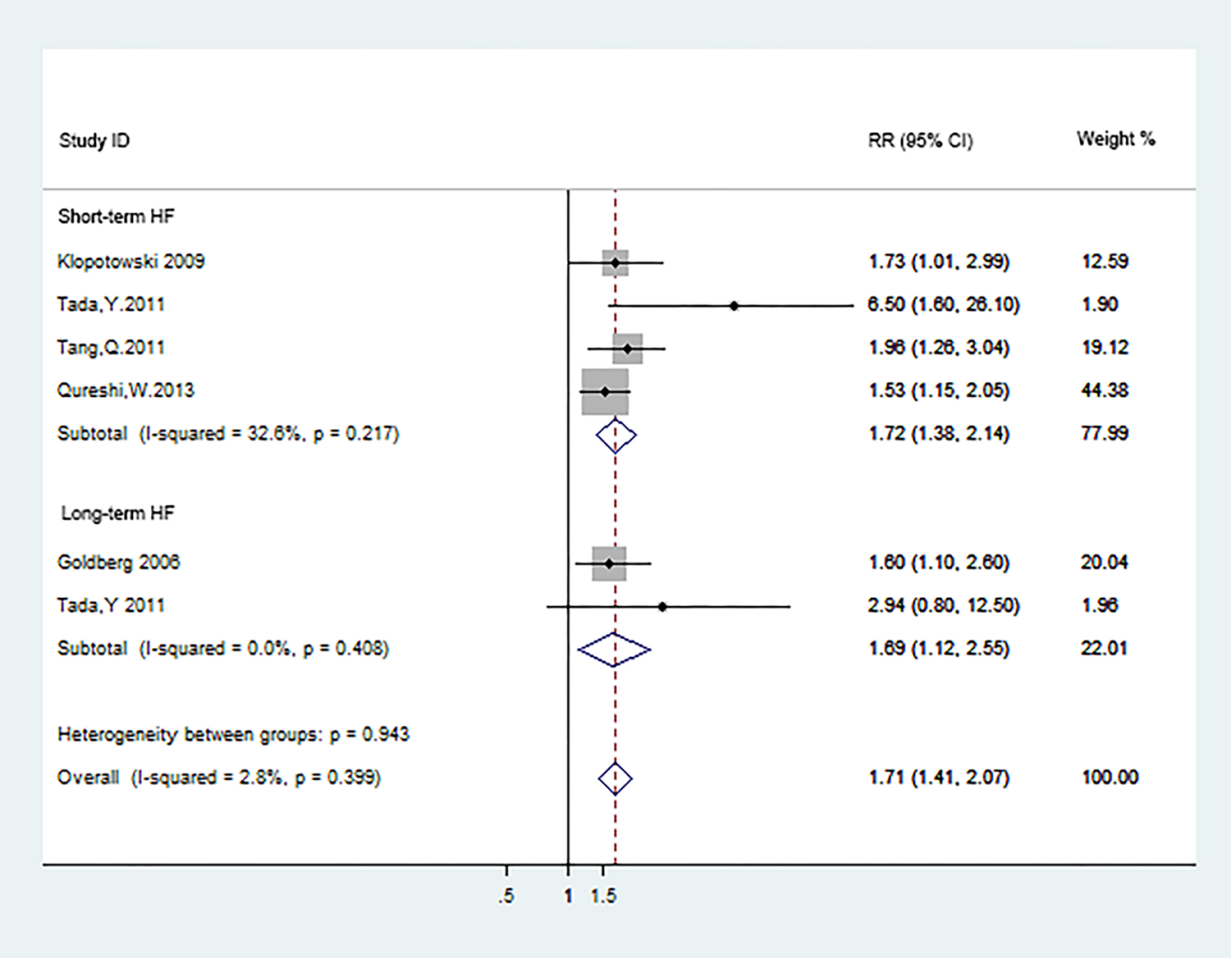
Heart failure in short- and long-terms.

For short-term heart failure in ACS, which defined as patients who had heart failure during the hospital stay or were rehospitalized for heart failure within 30 days of discharge, four studies [17,21,22,25] were enrolled in the meta-analysis. Given the post-MI LV systolic function may affect the independently association between hyponatremia and endpoint outcomes, we extracted the adjusted hazard ration from these documents to calculate the pooled hazard ration. We noted that ACS with hyponatremia was associated with the risk of short-term HF (RR: 1.72; 95%CI: 1.38–2.14; P<0.0001; Fig 7), regardless of LVEF. No statistically significant heterogeneity was observed (P = 0.217, I^2^ = 32.6%).

In terms of long-term HF in ACS, there only two studies[14,21] reported the adjusted effect estimates and 95%CI, the pooled analysis results indicated that ACS with hyponatremia tended to higher risk of HF during the follow-up(RR:1.69; 95%CI: 1.12–2.55; P = 0.012; Fig 7). Unimportant heterogeneity was observed (P = 0.408,I^2^ = 0.0%). Regardless of the starting time of heart failure, the whole pooled RR is 1.71 (95%CI: 1.41–2.07; P<0.0001; Fig 7), which suggested that hyponatremia could be a promising predictor for heart failure in ACS.

### Subgroup analyses

**Fig 8:**
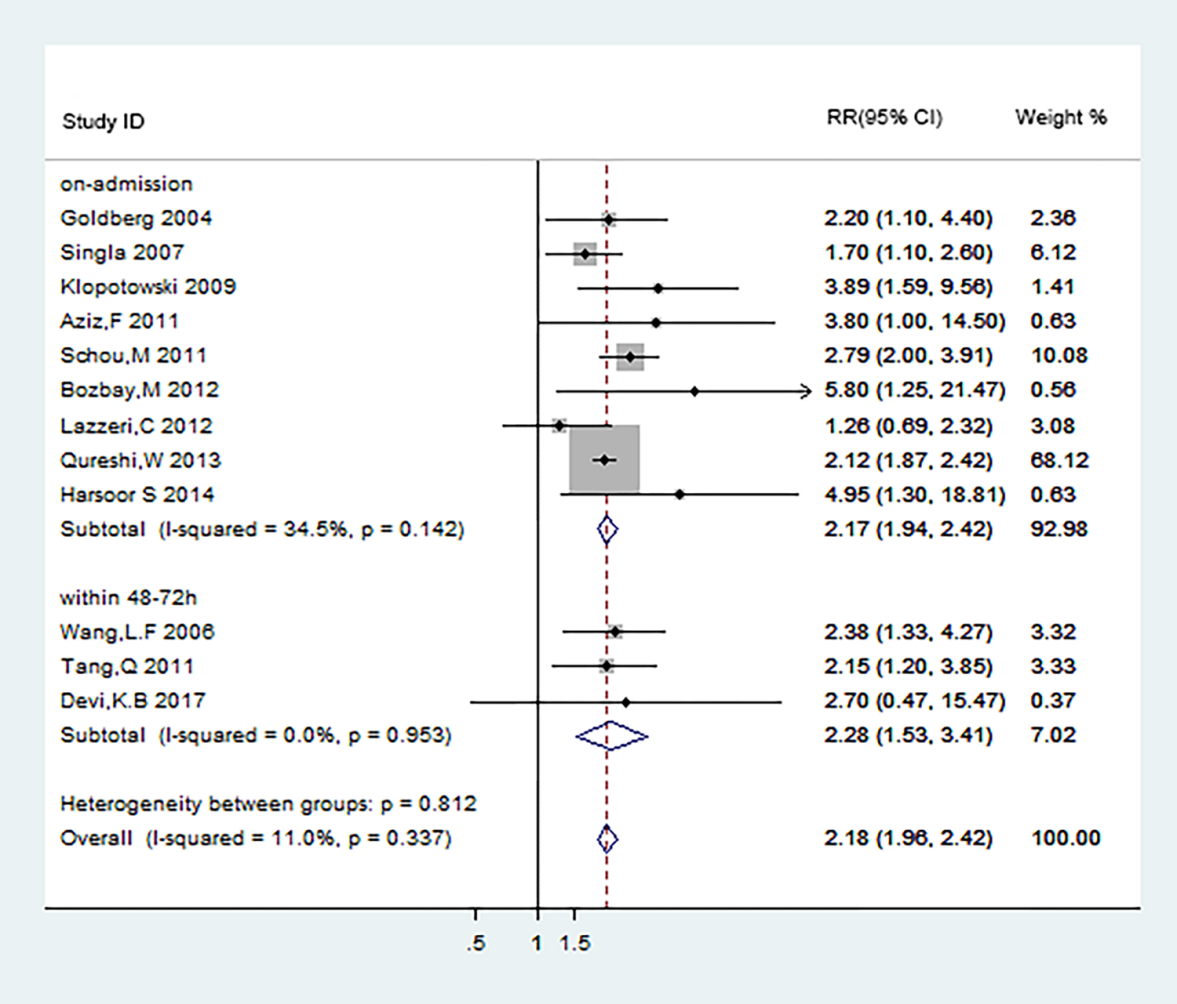
Subgroup analyses of short-term mortality according to different occurrence time of hyponatremia.

**Fig 9:**
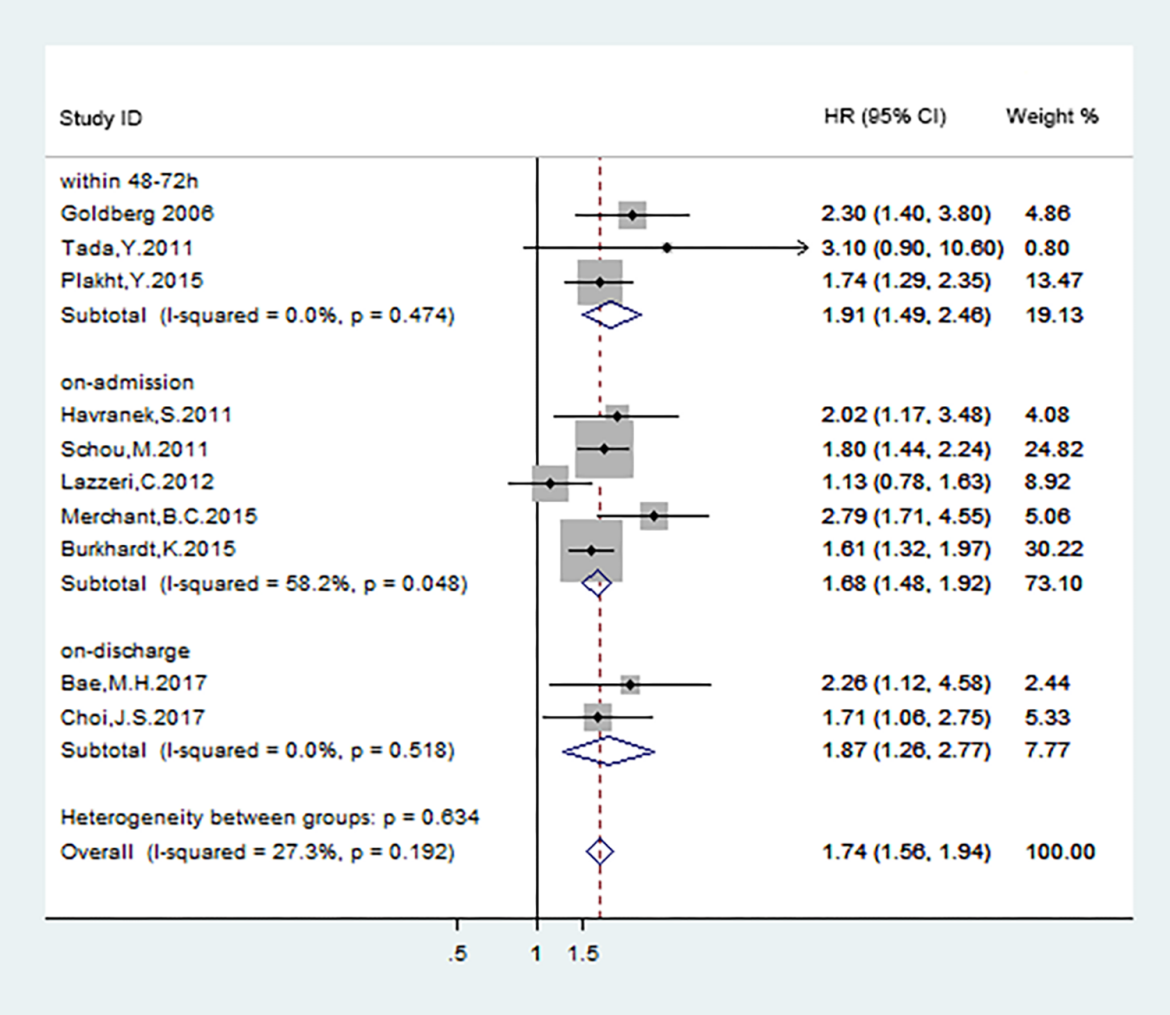
Subgroup analyses of long-term mortality according to different occurrence time of hyponatremia.

We did the subgroup analyses to find out the connection between the different occurrence time of hyponatremia in ACS patients and the short- and long-term mortality. The definition of hyponatremia in ACS patients was classified as hyponatremia at admission, hyponatremia within 48–72 hours after admission, and hyponatremia at discharge. The results were shown in Fig 8 and Fig 9.

Patients who were defined hyponatremia at admission and within 48–72 hours after admission both displayed significantly increased risk for short-term mortality (RR:2.17[95%CI: 1.94–2.42];RR:2.28[95%CI:1.53–3.41]) (Fig 8). Potential evidence of significant heterogeneity and publication bias were not detected. Data from 5[19,20,24,27,28], 3[14,21,29], 2[30,31]studies respectively were used to analyze the association of long-term mortality with hyponatremia at admission, within 48–72 hours after admission, and at discharge in ACS patients, the corresponding pooled HRs were 1.68(95%CI:1.48–1.92),1.91(95%CI:1.49–2.46) and 1.87(95%CI:1.26–2.77) (Fig 9). The risks of long-term mortality between subgroups showed no statistical significance. The results above indicated that hyponatremia occurred at different time during hospital in ACS patients were both associated with adverse clinical endpoint events.

**Fig 10:**
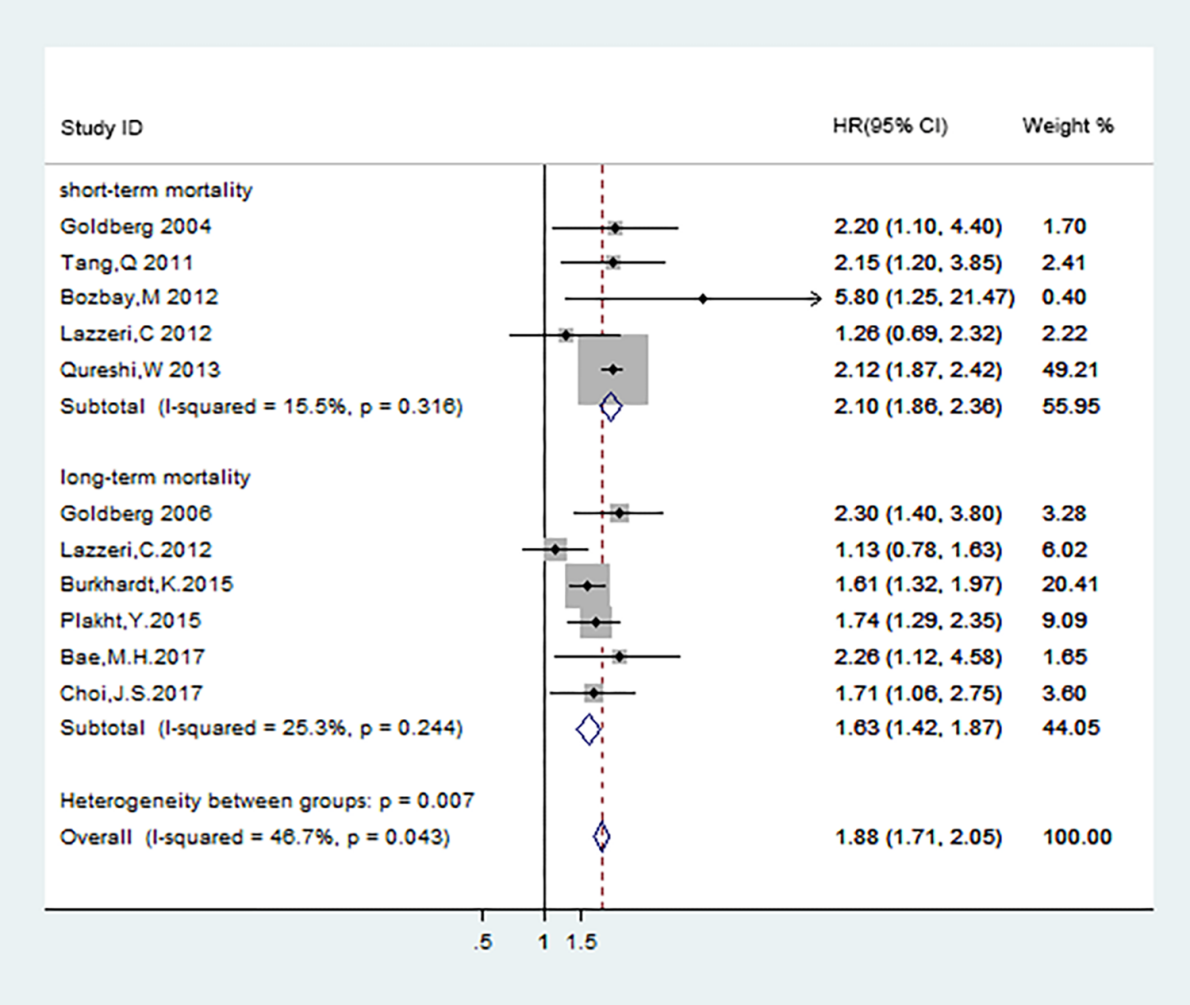
Subgroup analyses of short- and long-term mortality in patients after coronary intervention.

We also conducted subgroup analyses to determine whether the early coronary intervention may affect the independently association between hyponatremia and endpoint outcomes. There were 5[13,22–25] and 6[14,24,27,29–31] studies respectively reported the adjusted effect estimates in terms of short- and long-term mortality in ACS patients with coronary intervention, the corresponding pooled HRs were 2.10(95%CI:1.86–2.36;P<0.001) and 1.63(95%CI:1.42–1.87;P<0.001) (Fig 10).The results indicated that ACS with hyponatremia was an independent prognostic factor of adverse events in either the short- and long-term outcome, regardless of coronary intervention at the index hospitalization.

### Sensitivity analysis

We conducted sensitivity analysis to gauge the robustness of above results. When removing one study in sequence to see if a single study could make significant influence on the pooled effect sizes, the results were not significantly changed by removing anyone of the included studies (S1 Fig, short-term mortality; S2 Fig, subgroup analyses of short-term mortality; S3 Fig, long-term mortality; S4 Fig, heart failure).

## Discussion

Hyponatremia, a common electrolyte disturbance, is associated with poor prognosis in patients with acute heart failure [4, 5], liver cirrhosis [8], CKD [9, 10] and stroke [6, 7]. A meta-analysis of eighty-one studies found that hyponatremia happened in 17.4% of patient enrolled and is significantly associated with an increased risk of all-cause mortality [41].

Our meta-analysis of 20 cohort studies including 34,782 patients, which further investigated the prognostic value of hyponatremia in ACS patients, showed that hyponatremia is significantly associated with the increased risk of all-cause mortality within 30 days(RR: 2.18;95%CI: 1.96–2.42) and during the following-ups (HR: 1.74; 95%CI: 1.56–1.942). A negative association with short-term mortality was also observed for the subgroup analysis according to the sodium levels, the pooled RRs in the subgroups of 130–134 mmol/L and <130 mmol/L were 1.78 (95% CI: 1.34–2.35) and 3.64 (95%CI: 2.46–5.40) respectively, the lower serum sodium levels, the higher rates of the short-term mortality.

In early period of myocardial infarction, myocardial ischemia and anoxia could lead to intense sympathetic neural hyperactivity[42]. This results in profound stimulation of the sympathetic nervous system and renin-angiotensin-aldosterone system, causing peripheral vaso-constriction and redistribution of whole blood. Meanwhile, the levels of hormones such as catecholamines, AT II, aldosterone and AVP increased rapidly. Plasma AVP had a negative correlation with serum sodium levels in the patients with STEMI [21]. Elevated AVP increases the water permeability of distal convoluted tubule and collecting duct cells in the kidney, thus allowing water reabsorption. This occurs through increased transcription and insertion of water channels (Aquaporin-2) into the apical membrane of distal convoluted tubule and collecting duct epithelial cells, ultimately leading to hyponatremia. Tada, Y et all reported the plasma AVP level was significantly higher in patients who had a fatal outcome after AMI [21]. Possible causes to explanation are that elevated AVP promotes continued myocardial protein synthesis and fibroblast proliferation in mammalian myocardium, resulting in irreversible structural changes[43]. Hyponatremia in advanced CKD patients, who have no ability to concentrate urine in response to AVP, was still independently related with mortality after adjusting for other risk factors, indicating that hyponatremia may be directly toxic [43]. The level of serum sodium may impact the transmembrane potentials in cardiac cells, the formation of proteins and enzymes and muscle excitation[43]. On the other hand, patients who have the unfavorable prognosis in ACS are more ill, in those patients hyponatremia may be a biological marker of bad baseline condition [24].

Previous studies have shown that hyponatremia is an independent risk factor for poor prognosis of heart failure [4, 5], but the relationship is still unclear for patients after ACS without heart failure. In this meta-analysis, we summarized and analyzed four studies, which focused on the relationship of hyponatremia with the short-term heart failure, the pooled RR is 1.72(95%CI: 1.38–2.14), which suggested the presence of hyponatremia in patients with ACS may predict an increased risk of short-term heart failure. As for the long-term heart failure, Goldberg, A et all observed that hyponatremia on admission or developing during the first 72 hours of hospitalization in STEMI was independently associated with a higher incidence of post-discharge readmission for heart failure in long-term follow up[14], and Tada, Y et all also reported that early-developed hyponatremia could be a promising predictor of readmission due to heart failure in the long term, but not as a predictor of cardiac death[21]. After the meta-analysis of above studies, we concluded that hyponatremia is a predictor of heart failure in patients with ACS (RR: 1.71; 95%CI: 1.41–2.07), the potential mechanism may be similar to the influence of hyponatremia on prognosis in patients with congestive heart failure–the release of vasopressin, activation of the renin-angiotensin system and catecholamine production[11, 12].

There are also some limitations in the current meta-analysis which should be considered. First, among the 20 included studies, there are only two studies which reported data about the post-discharge readmission for heart failure in long-term follow-ups, this may result in potential publication bias, because positive results were prone to acceptance for publication than negative results. Second, the methods of calculation and extraction of data from survival curves in two studies [19, 21] might be less reliable, compared to those directly obtained from original article. Finally, due to less related data, we did not perform further subgroup analysis according to the duration of hyponatremia and the types of acute coronary syndrome.

In conclusion, despite some limitations mentioned above, we found that the level of serum sodium may be a predictive factor for patients with ACS. Based on the currently published literature, hyponatremia has a significant prognostic value for short- and long-term adverse events in patients after ACS. Thus, the dynamic monitoring of serum sodium levels may could help physicians to identify high risk ACS patients and to stratify risk for optimal management.

## Supporting information

S1 Fig Sensitivity analysis for studies on short-term mortality in ACS between hyponatremia and the normal controls. (TIF)

S2 Fig Sensitivity analysis for subgroup analyses of short-term mortality according to serum Na+ levels in ACS. *: represents the group of serum Na+ levels between 130-134mmol/L; **: represents the group of serum Na+ levels <130mmol/L.
(TIF)

S3 Fig Sensitivity analysis for studies on long-term mortality in ACS between hyponatremia and the normal controls. (TIF)

S4 Fig Sensitivity analysis for studies on heart failure in ACS between hyponatremia and the normal controls. *: represents the group of heart failure in short term.; **: represents the group of heart failure in long-term.
(TIF)

S1 File PRISMA checklist. (DOCX)
