# Supplementary figures and images for: Short- and long-term prognostic value of hyponatremia in patients with acute coronary syndrome: A systematic review and meta-analysis
Source: PLoS One. 2018 Mar 2;13(3):e0193857. doi: 10.1371/journal.pone.0193857 (PMC5834161; doi:10.1371/journal.pone.0193857)

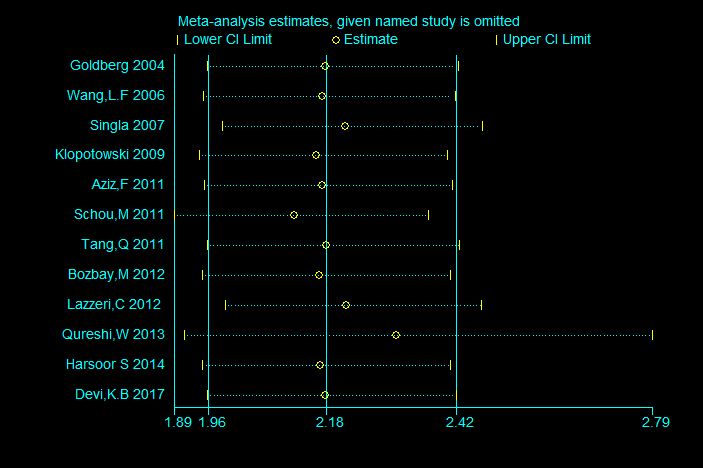

Supplement: S1 Fig — (TIF) [file pone.0193857.s001.tif]

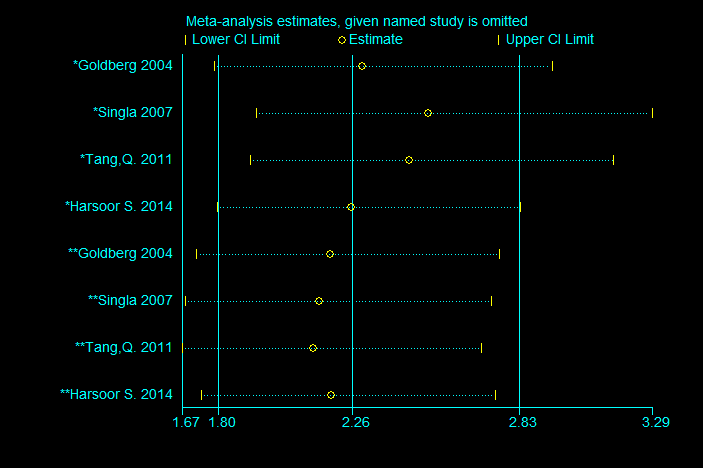

Supplement: S2 Fig — *: represents the group of serum Na+ levels between 130-134mmol/L; **: represents the group of serum Na+ levels <130mmol/L. (TIF) [file pone.0193857.s002.tif]

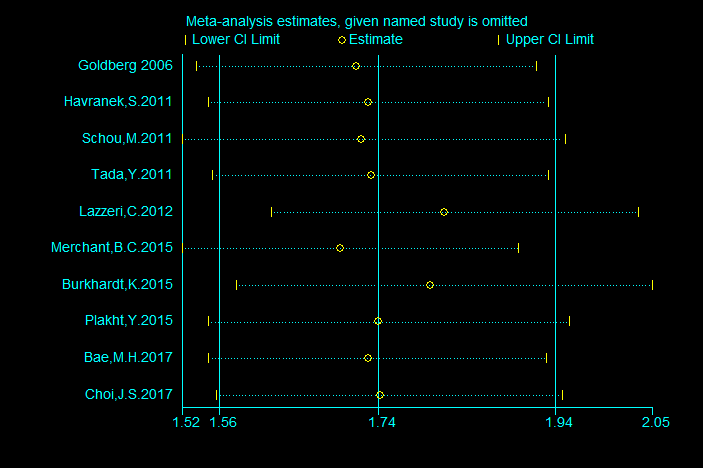

Supplement: S3 Fig — (TIF) [file pone.0193857.s003.tif]

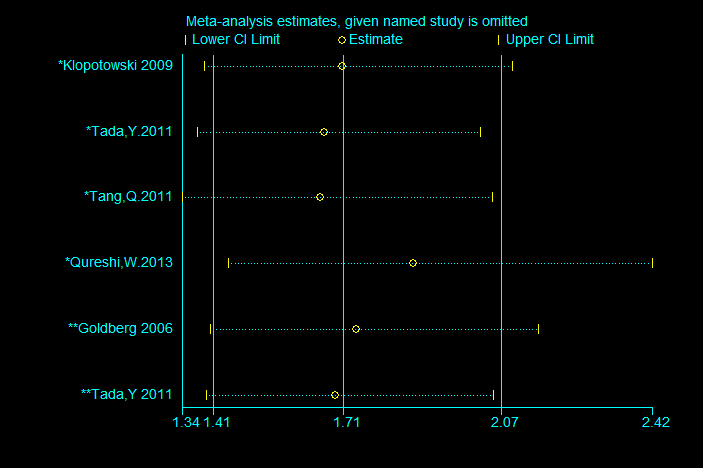

Supplement: S4 Fig — *: represents the group of heart failure in short term.; **: represents the group of heart failure in long-term. (TIF) [file pone.0193857.s004.tif]
